# Aneurism.—Report of a Case

**Published:** 1910-04

**Authors:** Harry Ainsworth

**Affiliations:** Thomasville, Ga.


### ANEURISM.—REPORT OF A CASE.

BY HARRY AINSWORTH, THOMASVILLE, GA.

An Aneurism is a saculated tumor, the cavity of which communicates with an artery; and, in some instances, also with a vein. They are classified as spherical, fusiform, and dissecting. A spherical Aneurism is one in which the tumor is well defined, its diameter being larger than that of the opening of the communication with the vessel.

In the fusiform aneurism there is a gradual and general dilitation of an artery in its entire circumference.

A dissecting Aneurism is one in which owing to pathological changes in the intima, the blood insinuates itself between the inner coat and the edventitia, dissecting the intima from the media and advntitia, and re-enters the vessel at a distant opening.

Aneurisms are further divide into true and false. The walls of the true are composed of the walls of the vessel from which they spring. The false are composed of inflammatory newly formed tissue.

*Cause.*—A true aneurism is always preceded by arteritis which results in degeneration of the walls of the vessel. Syphilis is the most usual cause.

It is well known that the large majority of true Aneurisms form at those points of the arterial system that are subjected to the greatest violence of the heart action, or muscular or mechanical pressure; such as the arch of the aorta or the popliteal arteries.

An Aneurism may in rare instances communicate with a vein: varicose Aneurism.

The direct communication of an artery and a vein without a sac is known as Aneurismal varix.

The false, or so-called diffuse Aneurism results from injury and the diffusion of blood into the periarterial tissues. The extravisation continues until the resistance of the surrounding tissues is equal to the pressure of the column of blood within the vessel. As a result, an inflammatory process of variable intensity, and usually non effective, is established, resulting in the formation of a limiting membrane or Aneurismal sac.

The prognosis varies under widely differing conditions. In general it is a grave affection, the gravity depending upon the location and character of the tumor, and the physical condition of the individual.

The symptoms of Aneurisms are in a great part local. A sense of unusual throbbing, pain more or less severe, and swelling in the line of the artery. It gives to the touch a tremor not easily described, but readily appreciated. The stechoscope conveys to the ear the peculiar bruit.

Left to nature the progress of an Aneurism is, with rare exception, to a fatal termination.

The treatment of an Aneurism is constitutional and local.

The constitutional treatment is directed toward the judicious support of the physical powers of the patient. The relief from pain, the production of a condition of the blood favorable to a deposit of fibrim in the tumor.

The constitutional measures alone offer little hope of a cure and are applicable only to the cases where the dangers of operative interference are sufficient to contra-indicate surgical procedure.

The local measures are directed to the mechanical control and arrest, either gradual or immediate, of the circulation in the sac.

The operations for Aneurism are varied and numerous, each one at times being popular.

The only one that I will try to describe is the matas. This method is to first control the blood supply; lay open the sac in its long axis; turn out all clots; wash out with gauze and normal saline solution; sew up all openings of small arteries, then by successive layers of fine cromacized catgut, completely obliterate the sac. Close up the incision either with or without drainage. This method is used whether the Aneurism is fusiform, saculated or spherical.

*Case.*—W. C.—Colored male, age thirty-six, admitted to City Hospital Dec. 12th. with gunshot wound of right thigh, upper third. The tract extending from inter to outer side. The femur being fractured. The wounds were cleaned and dressed and the fracture treated with sand bags with extension by pulleys and weights. Ten days later a plaster cast was applied. At this time on dressing the wound on inner side of thigh a pulsating area the size of the palm of the hand was left. There was no bulging of skin surface. This was easily diagnosed as an Aneurism. The plaster cast was nevertheless applied, deeming it advisable to let the femur heal first.

The treatment of Aneurisms had never before been up to me and I was rather at a loss. Among the surgeons asked in to see this case was one from one of our Northern Cities at the time visiting here. This appeared to be my opportunity, thinking only of the good of my patient. This Doctor was asked to operate. January 1st. was the date.

The patient, positively declining an anesthetic, spinal cocainization was resorted to, which was not satisfactory. The blood supply was not controlled, though the use of the tornequet was urged repeatedly upon the operator. In three minutes after the first incision there was a spurt of blood, and digital compression of the iliac against the brim of the pelvis was quickly resorted to; then the tornequet was applied.

From then on two operators were lost in the woods, where we hopelessly floundered. We did not know our heads from a hole in the ground. The idea of doing the matas operation was abandoned, and there was a vain effort to find and tie the femoral artery. After about two hours of fruitless endeavor the operation was terminated by sewing up the wound tightly, after first placing a guaze drainage.

I would like to comment on the supreme nerve displayed by the patient. He repeatedly refused an anesthetic, preferring the pain, which must have been severe. Another comment; there was also present at the ordeal a stout fellow practitioner from one of our Northern Cities. He was called upon to aid by using digital pressure on the femoral, which must have been quite a tax on one of his stoutness; at any rate he escaped as soon as possible and going to one of our drug stores for a drink of water, threw up his hands and exclaimed: "Whew, Good God, those boys like to have killed me."

The day following this attempt at surgery the leg became very oedemetous and considerable serum flowed from the gauze drainage.

This oedema was caused by pressure of the gauze packing upon the femoral vein.

This packing did not complete check the arterial blood supply, because the pulsation of the dorsalis pedes artery was constantly recognized.

After the lapse of a week the gauze drainage was each day pulled out about an inch.

The afternoon of January 11th, there was a hurry call to the Hospital. The message was, "Your nigger is bleeding to death." On reaching the Hospital it was found that the hemorrhage had been stopped by the nurse applying a tornequet high up about the thigh. As soon as the operating room could be got ready the patient was anesthetized with ether. The previous incision was reopened and pus was found in abundance. This was removed by irrigation. The femoral artery was found and litigated in two places about two and one-half inches apart. The wound was packed with gauze, no sutures being taken. The patient was placed in bed, well wrapped up, and hot water bags packed about the leg. For several days the patient was in a very critical condition, running a temperature of 102 to 103, pulse rate 130 to 140. The oedema of the leg immediately began to grow less, and in four days the leg was of normal size and quiet warm, showing that the collateral circulation had saved the day. The wound has now almost entirely healed and the patient up and walking about on crutches. "What luck some of us have."

